# Generalized Ligamentous Laxity; a Parameter Should not to be Forgotten in Preoperative Planning of Adolescent Idiopathic Scoliosis

**DOI:** 10.5812/ircmj.2554

**Published:** 2012-11-15

**Authors:** Ebrahim Ghayem-Hasankhani, Farzad Omidi-Kashani

**Affiliations:** 1 Orthopedic Research Center, Emam Reza Hospital, Faculty of Medicine, Mashhad University of Medical Sciences, Mashhad, Iran

**Keywords:** Idiopathic, Scoliosis, Ligaments, Surgical Procedures, Operative

## Abstract

**Background:**

Many factors effect on management (surgical and nonsurgical) of adolescent idiopathic scoliosis (AIS).

**Objectives:**

The purpose of this study was evaluation of the effects of generalized ligamentous laxity (GLL) on surgical treatment of AIS.

**Materials and Methods:**

72 patients with AIS were studied between 2002 and 2009. 24 cases (33.4%) were placed in group A (normal) while 48 patients (66.6%) with GLL in group B. Our threshold for adding anterior approach was a curve which could not be corrected to < 50° on the supine lateral bending view.

**Results:**

The mean age and follow up period were 16.4 (12-22 years) and 3.8 (2-6.5 years), respectively. In the first group, 12 (50%) were operated with combined anterior and posterior approaches while in the later; there were only 6 (12.5%). Curve correction was 73.3% in patients with GLL and 57.1% in patients without it. Both of these differences were significant statistically (P = 0.001).

**Conclusions:**

In preoperative planning of surgical treatment of AIS, GLL is an important factor. In this special group of patients due to much more flexibility, relatively larger scoliotic curves can be safely treated by single posterior approach.

## 1. Background

AIS is one of the most common spinal deformities with no definite etiology, although there are some probable causes reported to be important in creating and exaggerating it (1, 2). The deformity can be treated with observation alone, bracing or surgery. In surgical treatment, the base of the remedy is on the spinal arthrodesis. In acquiring a successful treatment, it should be tried to fuse as little motion segment as possible and also to operate as few and less aggressive approaches as possible (3).

One of the indices that thought to be important in preoperative planning of this disorder is scoliotic curve flexibility and one of the factors that is critical in curve flexibility besides Cobb angle, curve location, sex and age; it seems to be GLL (4-6). According to our knowledge, the effect of GLL on the surgical treatment of AIS has not been directly investigated yet.

## 2. Objectives

In this study we tried to analyze the effects of GLL in the surgical treatment of AIS.

## 3. Materials and Methods

In this prospective cohort study, we studied 83 surgically treated cases with AIS between May 2002 and January 2009. Preoperatively, in all the patients standing radiographs and supine lateral bending views and in selected cases, total spine MRI and pulmonary function tests were acquired. All the patients assigned the informed consent.

Flexibility and correction percentages were acquired as below:

Flexibility percentage (%) = (upright Cobb° - supine bending Cobb°)/upright Cobb° × 100

Correction percentage (%) = (upright Cobb° postop - upright Cobb° preop)/ upright Cobb° preop × 100

All participants were examined for GLL using the adapted Beighton-Horan scale (7). This laxity assessment, was first devised by Carter and Wilkinson in 1964 and later modified by Beighton et al in 1973 (8), measures the following five elements:

1-Passive opposition of the thumb to the flexor aspect of the forearm (1 point per hand)

2-Passive hyperextension of the 5th metacarpophalangeal joint > 90˚ (1point per hand)

3-Hyperextension of the elbows >15˚ (1 point per arm)

4-Hyperextension of the knees (1 point per leg)

5-Forward flexion of the trunk with knees extended and palms flat on floor (1 point)

All elements are added together to give an overall ligamentous laxity score ranging from 0 (tight) to 9 (hyperlax). After the assessment, we arbitrary stratified the participants into one of two groups: group A (tight group; scores 0–4) and group B (loose group; scores 5–9).

Our threshold for adding anterior approach was a curve which can’t be corrected to < 50° on the supine bending lateral view. We routinely instrumented the spine with combined hooks and pedicular screws for segmental fixation. The patients with less than 2 years following up were excluded from the study.

Statistical analysis was performed by the Mann-Whitney test for comparing scoliotic curves with the SPSS statistical software (Version 15, Chicago, IL, USA). P < 0.05 was considered statistically significant.

## 4. Results

Initially, we had 83 patients fulfilled the criteria of the study, but later 8 patients cannot be accessed and followed up and therefore they were exited from it. Another 3 cases were also excluded due to the underlying syrinx. Finally, we considered 72 cases (18 male; 25%, and 54 female; 75%). The mean age and follow up period were 16.4 (12-22 years) and 3.8 (2-6.5 years), respectively.

24 cases (33.4%) were placed in group A while 48 patients (66.6%) in group B. In the first group, 12 (50%) were operated with combined approaches while in the later; there were only 6 (12.5%). Correction percentage in both groups was depicted in Table 1. Postoperative complications were not remarkable.

**Table 1:** Correction percentage of the scoliosis in our patients

Groups	Preoperative Curve, Mean ± SD	Flexibility Percentage, Mean ± SD	Postoperative Curve, Mean ± SD	Correction Percentage
**A**	71.17 ± 14.98	31.2 ± 13.3	30.52 ± 9.15	58.1%
**B**	66.73 ± 12.33	41.4 ± 16.5	17.84 ± 4.48	73.3%
**P value**	0.52	0.01	0.013	0.001

## 5. Discussion

The main goal of surgery in AIS is prohibition of the curve aggravation. The secondary gains are correction of the curve as safe as possible, balancing the trunk decompensation (if present), rib hump elimination and shoulders leveling; while fusing as few vertebrae as possible. In acquiring these goals, numerous factors including surgical technique, type of the instrumentation and deformity, maturity, sex, Cobb angle, location and flexibility of the curve should be taken into account (5, 9-11).

The prevalence of GLL in our study was 66.6% and was surprisingly high relative to some other studies like the one conducted by Stewart and Burden (12). They found a prevalence of 24% with a criteria > 4/9 for GLL. In this study we cannot justify it but geographic differences among the patients or other unknown parameters may play some role. Therefore in preoperative planning of every patient who is candidate for AIS surgery, GLL as a good prognostic index should be checked carefully. In this special group of patients due to much more flexibility, relatively larger scoliotic curves can be safely treated by single posterior approach.
